# Enhanced peptide quantification using spectral count clustering and cluster abundance

**DOI:** 10.1186/1471-2105-12-423

**Published:** 2011-10-28

**Authors:** Seungmook Lee, Min-Seok Kwon, Hyoung-Joo Lee, Young-Ki Paik, Haixu Tang, Jae K Lee, Taesung Park

**Affiliations:** 1 Department of Statistics, Seoul National University, Korea; 2 Interdisciplinary program in Bioinformatics, Seoul National University, Korea; 3 Department of Biochemistry, Yonsei Proteome Research Center and Biomedical Proteome Research Center, Yonsei University, Korea; 4 Center for Genomics and Bioinformatics, Indiana University, USA; 5 Department of Public Health Sciences, Division of Biostatistics, University of Virginia School of Medicine, USA

## Abstract

**Background:**

Quantification of protein expression by means of mass spectrometry (MS) has been introduced in various proteomics studies. In particular, two label-free quantification methods, such as spectral counting and spectra feature analysis have been extensively investigated in a wide variety of proteomic studies. The cornerstone of both methods is peptide identification based on a proteomic database search and subsequent estimation of peptide retention time. However, they often suffer from restrictive database search and inaccurate estimation of the liquid chromatography (LC) retention time. Furthermore, conventional peptide identification methods based on the spectral library search algorithms such as SEQUEST or SpectraST have been found to provide neither the best match nor high-scored matches. Lastly, these methods are limited in the sense that target peptides cannot be identified unless they have been previously generated and stored into the database or spectral libraries.

To overcome these limitations, we propose a novel method, namely *Quantification method based on Finding the Identical Spectral set for a Homogenous peptide *(Q-FISH) to estimate the peptide's abundance from its tandem mass spectrometry (MS/MS) spectra through the direct comparison of experimental spectra. Intuitively, our Q-FISH method compares all possible pairs of experimental spectra in order to identify both known and novel proteins, significantly enhancing identification accuracy by grouping replicated spectra from the same peptide targets.

**Results:**

We applied Q-FISH to Nano-LC-MS/MS data obtained from human hepatocellular carcinoma (HCC) and normal liver tissue samples to identify differentially expressed peptides between the normal and disease samples. For a total of 44,318 spectra obtained through MS/MS analysis, Q-FISH yielded 14,747 clusters. Among these, 5,777 clusters were identified only in the HCC sample, 6,648 clusters only in the normal tissue sample, and 2,323 clusters both in the HCC and normal tissue samples. While it will be interesting to investigate peptide clusters only found from one sample, further examined spectral clusters identified both in the HCC and normal samples since our goal is to identify and assess differentially expressed peptides quantitatively. The next step was to perform a beta-binomial test to isolate differentially expressed peptides between the HCC and normal tissue samples. This test resulted in 84 peptides with significantly differential spectral counts between the HCC and normal tissue samples. We independently identified 50 and 95 peptides by SEQUEST, of which 24 and 56 peptides, respectively, were found to be known biomarkers for the human liver cancer. Comparing Q-FISH and SEQUEST results, we found 22 of the differentially expressed 84 peptides by Q-FISH were also identified by SEQUEST. Remarkably, of these 22 peptides discovered both by Q-FISH and SEQUEST, 13 peptides are known for human liver cancer and the remaining 9 peptides are known to be associated with other cancers.

**Conclusions:**

We proposed a novel statistical method, Q-FISH, for accurately identifying protein species and simultaneously quantifying the expression levels of identified peptides from mass spectrometry data. Q-FISH analysis on human HCC and liver tissue samples identified many protein biomarkers that are highly relevant to HCC. Q-FISH can be a useful tool both for peptide identification and quantification on mass spectrometry data analysis. It may also prove to be more effective in discovering novel protein biomarkers than SEQUEST and other standard methods.

## Background

The main objective of functional proteomics analysis is often to estimate changes in the amount of proteins found in complex biological systems, in response to physiological and clinical factors such as cell development, disease progression, or drug treatment. In particular, one of the key issues in proteomics research based on tandem mass spectrometry (MS/MS) is the identification of protein species and the characterization of their expression changes in normal and disease samples. Three analysis techniques are often required in an MS/MS study: expressed peptide identification, target protein characterization, and quantification [1]. For hundreds to tens of thousands of fragment ion spectra generated, the assignment of the fragment ion spectra to peptide sequences, the identification of proteins represented by each peptide, and the estimation of their abundances in the analyzed sample require complex computations and still remain as high statistical challenges [2].

Quantification of protein expression using mass spectrometry (MS) is often required for the discovery of protein biomarkers associated with cancer, their response to stimuli, cell signalling cascades and the function of cell cycle-promoting proteins, and various biomedical investigations [3]. Two categories of quantification methods for MS data have been used: stable isotope labelling quantification and label-free quantification [2].

Several stable isotope-based quantification methods have been introduced based on different labelling reagents that can be chemically bound to peptides [4]. It is, however, difficult to simultaneously quantify the amount of proteins/peptides in multiple samples because of the limited number of labelling reagents available [5]. Moreover, current practical applications can typically quantify, at most, a few hundreds of peptides, measuring relative expression values of each pair of contrasting samples. Furthermore, the high costs of labelling reagents make these quantification methods difficult to be commonly applied for the characterization of the global proteome.

On the other hand, label-free quantification, which does not require the use of a stable isotope labeling, has the advantages of low cost and simplicity. Currently, two label-free methods are available to measure expression levels of peptides: spectra counting and spectra feature analysis. The spectral counting method can estimate the peptide expression levels by means of spectrum counting (from MS/MS data) or through the estimation of the integrated ion intensities [6,7]. The spectral feature analysis method quantitatively determines the peptide expression levels by comparing three-dimensional patterns (retention time, m/z and intensity) between different samples [8-13].

However, these label-free quantitative methods have two main shortcomings. The first limitation is due to numerous false-positive discriminative peptides, which are the result of the chromatographic variability between LC-MS experiments. In the analysis of the spectra features, after finding two candidates with same MS1 retention time and m/z, the difference in their MS1 intensities is used to define the peptide levels. Therefore, spectra feature analysis requires stringent reproducibility [3,8] and additional pre-processing of the LC normalization or retention time alignment [14,15].

The second limitation is that spectra counting cannot be performed without peptide identification because the relative peptide levels can be quantified only after peptide identification. In peptide identification, MS/MS spectra are verified using a database searching algorithm or spectral library searching algorithm such as SEQUEST, MASCOT, or SpectraST. Specifically, database search algorithms calculate score functions to compare the experimental MS/MS spectra with theoretical MS/MS spectra of peptides derived from protein sequence databases. The pool of theoretical MS/MS spectra is restricted by user-specified criteria such as mass tolerance, proteolytic enzymes, and the types of post-translational modification [2,16]. A number of spectra may not be assigned to the correct peptides for diverse reasons, including deficiencies of the scoring scheme implemented in the database search tools, sequence variations (e.g., single nucleotide polymorphisms, SNPs), omissions in the database searched, post-translational or chemical modifications of the peptide analyzed, and the observation of genomic sequences that are not anticipated (e.g., splice forms, somatic rearrangement, and processed proteins) [17]. For all these reasons, a large number of important peptides may be lost during the database search.

Instead of matching acquired MS/MS spectra against theoretically predicted spectra, MS/MS spectra can also be assigned to peptides by matching those in a spectral library. The spectral library is compiled from a large collection of experimentally observed MS/MS spectra identified in previous experiments [18]. Generally, a set of spectra of known peptide sequences is collected into a library and used as a reference. The experimental spectrum may be identified by a similar match in the library. However, this method can only be identified when spectra were observed previously and entered into the library. So, these library searching methods are well suited for targeted proteomics, in which one seeks not to discover previously unseen peptides, but rather limited to finding and quantifying expected peptides of interest in the sample [19].

To overcome these limitations of label-free quantification methods, we propose a novel spectral counting method to estimate a peptide's abundance by counting MS/MS spectra, comparing and clustering all experimentally observed spectra. This approach has several advantages. First, because the same peptide may be fragmented multiple times or repeatedly observed at different time points from an MS/MS run, multiple spectra may be extracted for the same peptides. In other words, duplicated spectra are ubiquitous in large-scale proteomics data [20]. Our method thus attempts to identify and group all the duplicate spectra, which allows us to quantify the amount of peptide found in complex biological systems without searching through the databases or using LC normalization.

**Figure 1:**
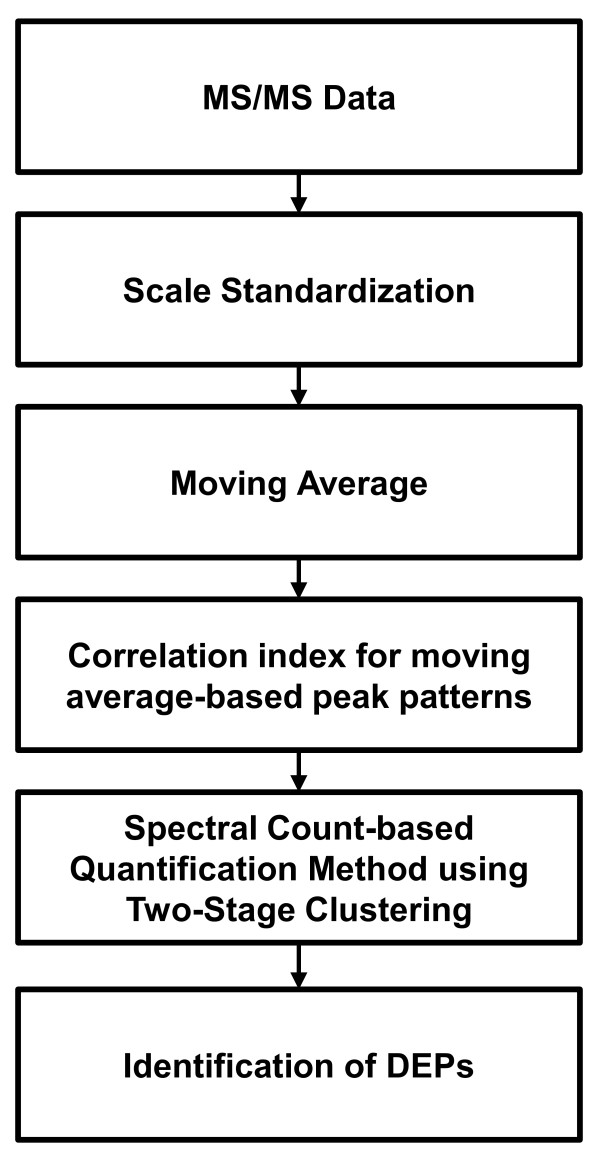
**Work flow chart**. This figure shows a flow schematic of the analysis process performed by Q-FISH algorithm

For the given spectra, our method, referred to as the *Quantification method derived by Finding the Identical Spectra set for a Homogenous peptide *(Q-FISH) employs a two-stage clustering algorithm to determine whether they are from the same peptides with homogeneous spectral patterns. The Q-FISH algorithm employs two similarity measures: the difference between two precursor ions and the correlation coefficient of moving window averages. Subsequently, the algorithm clusters spectra from the same peptide through all plausible pair-wise comparisons. By counting the spectra of each cluster set of peptides, we can estimate the amount of peptides. Figure 1 summarizes the workflow of the proposed Q-FISH algorithm.

Our proposed algorithm was applied to identify differentially expressed peptides from a real data obtained during a Nano-LC-MS/MS experiment performed on human HCC and normal liver tissue samples.

## Results & Discussion

We introduced and tested the so-called Q-FISH algorithm to identify and quantify the amount of all expressed peptides from an MS/MS dataset by clustering and counting spectra with homogeneous spectral patterns. In order to test our algorithm, we performed a Nano-LC MS/MS experiment with triplicated human hepatocellular carcinoma and normal liver tissue samples. For a total of 44,318 MS/MS spectra obtained through three MS/MS analysis for two samples, Q-FISH yielded 14,748 clusters. More specifically, 5,777 clusters were identified only in the hepatocellular carcinoma (HCC) sample, 6,648 clusters only in the normal sample, and 2,323 clusters in both HCC and normal samples. For the purpose of comparison, we also implemented SEQUEST and SpectraST to identify peptides. However, only 4,824 of 44,318 spectra were identified using SEQUEST, and a total of 1,326 peptides from the experimental spectra. Generally, most database search algorithms including SEQUEST assign specific experimental spectra to peptides by comparing the experimental data with theoretical spectra generated from the peptide sequence. It should be noted that neither the best match nor a high search score may not be a true match, especially for novel protein targets. Therefore, many peptides could be misidentified, or not be identified, unless they were previously generated and stored into the database sequence. In our experiments, a large number of experimental spectra (89.12%, namely 39,494 of a total of 44,318 spectra) could not be used for the peptide identification using SEQUEST. On the other hands, 5,549 spectra and 3,295 peptides could be identified using SpectraST. That is, a large number of spectra still could not be used for the peptide identification by SpectraST (87.48%, namely 38,769 of a total of 44,318 spectra). On the other hand, our proposed method directly compares all observed experimental spectra to discover differentially expressed peptides without a loss of observed spectra.

**Figure 2:**
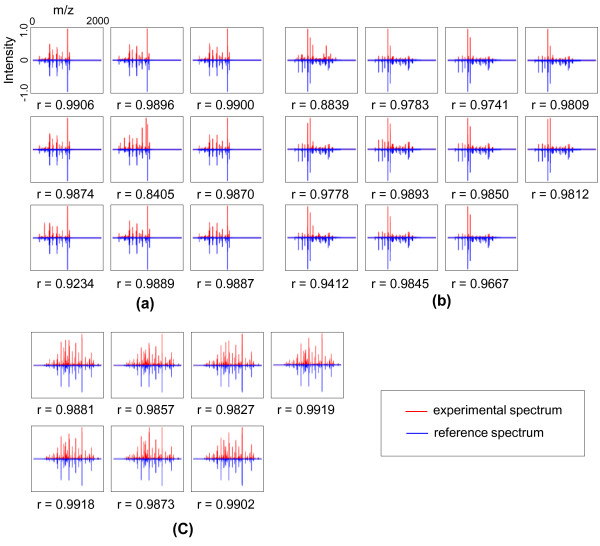
**Pattern-plots of reference spectrum and experimental MS/MS spectra in clustered spectral sets**. This figure shows pattern-plots the of the experimental MS/MS spectra with plotted using positive intensities (upper part) and the reference spectrum using negative intensities (lower part). Then, (a) all of nine spectra were identified as a same peptide, while (b) two of the eleven spectra are not identified by SEQUEST and (c) four of the seven spectra were only identified by SpectraST, although pattern-plots are very similar.

The standardized intensities of the experimental spectra plotted in Figure 2 are characterized by positive intensity values (upper part) and the reference spectrum plotted using negative intensity values (lower part). Specifically, Figure 2(a), which illustrates an example of one cluster with nine similar spectra, shows spectral patterns of the MS/MS spectra as well as the reference spectrum for clustered spectral set. The overall patterns look quite similar and all nine spectra pairs seem to have almost identical patterns. Table 1 shows the search results returned by SEQUEST and SpectraST. Subsequently, in the case of spectral set S366006, nine spectra were identified by means of the same peptide sequence, "SIFSAVLDELK" in the SEQUEST and SpectraST with XCorr above 1.97. In addition, a reference spectrum for the clustered spectral set was identified as the peptide sequence, "SIFSAVLDELK" with a SEQUEST score, XCorr = 2.96. This analysis reveals that these spectra can be regarded as the spectra of a homogenous peptide. In other words, each cluster could be expected to be composed of spectra from the same peptide.

**Table 1:** Results of SEQUEST & SpectraST for spectra in clustered spectral sets

Spectral Set ID	Sample	Sequence	XCorr	RT	precursor ion	precursor intensity	SpectraST
S366006	HCC-3	SIFSAVLDELK	2.35	6096.18	1223.4	25828.2	1
	Normal-1	SIFSAVLDELK	2.38	6144.00	1222.6	12823.4	1
	Normal-1	SIFSAVLDELK	2.12	6197.20	1224.2	385800.0	1
	Normal-1	SIFSAVLDELK	2.30	6248.89	1224.2	145284.0	1
	Normal-2	SIFSAVLDELK	1.99	6278.55	1223.4	6218.5	1
	Normal-2	SIFSAVLDELK	2.35	6341.52	1222.3	14101.8	1
	Normal-2	SIFSAVLDELK	1.98	6441.91	1223.1	2800.2	1
	Normal-3	SIFSAVLDELK	2.56	6149.98	1224.4	421560.0	1
	Normal-3	SIFSAVLDELK	2.37	6154.65	1222.3	23456.6	1

S1157004	Normal-1	VDFPQDQLTALTGR	2.77	3724.74	1565.1	106099.0	1
	Normal-2	VDFPQDQLTALTGR	2.33	3647.59	1562.4	143286.0	1
	Normal-1	VDFPQDQLTALTGR	2.46	3779.98	1562.2	75465.1	1
	HCC-3	VDFPQDQLTALTGR	2.10	3854.18	1562.3	34323.8	1
	Normal-2	VDFPQDQLTALTGR	2.07	3695.20	1562.7	159244.0	1
	Normal-3	VDFPQDQLTALTGR	2.07	3825.73	1562.4	69159.3	1
	Normal-3	VDFPQDQLTALTGR	2.24	3775.23	1562.9	71196.3	1
	HCC-1	VDFPQDQLTALTGR	2.02	3930.66	1562.2	25175.7	1
	HCC-2	VDFPQDQLTALTGR	1.95	3977.91	1562.2	12849.6	1
	Normal-2	M#WLSSMCSMRSAR	1.29	3629.94	1564.7	86816.8	1
	HCC-1	VDFPQDQLTALTGR	2.20	3907.36	1564.2	19403.5	1

S65002	HCC-1	EILVGDVGQTVDDPYATFVK	3.81	4619.64	1084.4	21166.7	1
	HCC-3	EILVGDVGQTVDDPYATFVK	3.70	4573.01	1084.7	46939.4	X
	HCC-3	EILVGDVGQTVDDPYATFVK	2.32	4579.55	1083.5	22077.0	1
	Normal-1	EILVGDVGQTVDDPYATFVK	2.87	4516.45	1084.0	26598.5	1
	Normal-2	EILVGDVGQTVDDPYATFVK	4.08	4461.41	1084.5	19416.7	X
	Normal-2	EILVGDVGQTVDDPYATFVK	3.49	4514.74	1084.7	91100.7	X
	Normal-3	EILVGDVGQTVDDPYATFVK	3.37	4548.32	1084.5	23254.4	1

These spectra were clustered by the proposed Q-FISH algorithm. In the case of spectral set S366006, all spectra in spectral set were identified as a same peptide sequence "SIFSAVLDELK" by both of SEQUEST and SpectraST, while two spectra in S1157004 are not identified by SEQUEST (XCorr < 2.11). Also, all spectra in S65002 are identified by SEQUEST with high scores, while four spectra were only identified by SpectraST. If we relied only on SEQUEST or SpectraST, these spectra in S1157004 or S65002 would be excluded.

Similarly, Figures 2(b) and 2(c) show spectral patterns for the reference spectrum and the experimental spectra of a single cluster. It should be noted that the overall patterns look quite similar and all spectra pairs are characterized by high correlation coefficients. However, while all spectra in S1157004 could be identified by SpectraST, two out of the eleven spectra could not be identified by SEQUEST, as shown Table 1. On the contrary, all spectra in S65002 are identified by SEQUEST with high scores, while three spectra could not be identified by SpectraST. In other words, if we relied only on the conventional peptide identification such as SEQUEST or SpectraST, these spectra would have been excluded despite the similar peak patterns. On the other hand, our Q-FISH algorithm was able to include these spectra without a loss of information.

**Figure 3:**
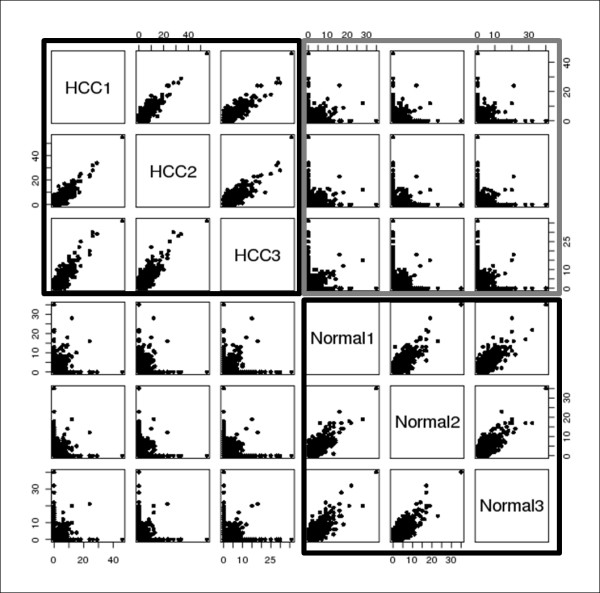
**Scatter plot between different samples and within replicated samples**. This figure represents the scatter plot with the number of spectra in clustered sets obtained through the replicated experiments on HCC and normal tissue samples, respectively. Then, two black boxes show the relationships of the number of spectra in replicated each HCC and normal samples, while a gray box represents the relationships of the number of spectra in clustered sets between HCC and normal samples.

In this study, we were interested in identifying proteins and characterizing their differential expressions in normal and HCC samples. Hence, we first focused on the 2,323 clusters, which were observed in both samples. Figure 3 and Table 2 show a scatter plot and a correlation matrix with the number of spectra in the same cluster, which were obtained through the replicated experiments on HCC and normal tissue samples, respectively. It is worth noting that the number of spectra in the same cluster showed high correlations (0.7178~0.8315), while the number of spectra for different samples showed weak correlations (0.0654~0.1549). For a given spectral set, the reference spectrum was estimated by averaging the relative intensities of the spectra. Consequently, the reference spectrum corresponds to the number of expressed spectra in the normal and HCC samples. We computed the false clustering rate (FCR) on the 2,323 clusters shared by the HCC and normal samples. Among these clusters, 1,571 clusters had FCRs smaller than 0.05. Our next step was to perform a beta-binomial test to isolate differentially expressed peptides (DEPs) [21]. The result showed that only 84 out of the 1,571 reference spectra were characterized by different spectral counts between the HCC and normal tissue samples. Also, 5,777 clusters were observed only in the HCC sample and 6,648 clusters only in the normal sample by Q-FISH. Among these clusters, 1,571 and 1,556 clusters, respectively, had FCRs smaller than 0.05.

**Table 2:** Correlation matrix and the number of shared spectral clusters between different samples and within replicated samples

	HCC1	HCC2	HCC3	Normal1	Normal2	Normal3
HCC1	1.0000^a^ (4,319)^b^	0.8315 (2,117)^c^	0.8125 (2,142)^c^	0.1549 (1,108)^c^	0.0828 (929)^c^	0.1088 (1,022)^c^
HCC2		1.0000 (4,144)^b^	0.8048 (2,032)^c^	0.1232 (1,025)^c^	0.0654 (894)^c^	0.0899 (947)^c^
HCC3			1.0000 (4,461)^b^	0.1394 (1,144)^c^	0.0654 (947)^c^	0.0911 (1,061)^c^
Normal1				1.0000 (4,710)^b^	0.7178 (2,280)^c^	0.7449 (2,286)^c^
Normal2					1.0000 (4,863)^b^	0.7302 (2,128)^c^
Normal3						1.0000 (4,560)^b^

^a^: correlation coefficient, ^b^: # of spectral clusters, and ^c^: # of shared spectral clusters,

This table shows correlation matrix with number of spectra in same cluster between different samples and within replicated samples. The number of spectra in the same cluster within replicated samples showed high correlations, while the number of spectra between different samples showed weak correlations.

In order to compare the performance of Q-FISH with the spectral counting method by SEQUEST, we used the human liver data and validated the results through literature search. For the human liver data, Q-FISH provided 1571 differentially expressed clusters for HCC sample and 1556 for normal sample, among which 57 and 99 clusters were identified by SEQUEST in HCC and normal samples, respectively. On the other hand, SEQUEST provided 93 and 145 peptides for HCC and normal tissue samples, respectively. Among the 57 identified clusters in HCC samples, 37 clusters were found to be over-expressed by Q-FISH; 20 peptides/clusters were overlapped by Q-FISH and SEQUEST. On the other hands, 73 peptides were identified only by SEQUEST. 49 peptides/clusters were identified as over-expressed by both Q-FISH and SEQUEST in normal sample. Also, 50 and 96 peptides/clusters were identified as over-expressed only by Q-FISH and SEQUEST, respectively.

We compared two results through literature search. We assumed that it is a true match if a peptide was reported in a previous literature in cancer. While there is a certain degree of uncertainty for reported protein biomarkers, this assumption is not biased to any of the two methods and allowed us to statistically compare their performance. For examples, alpha-2-macroglobulin (A2M) annotated by "VSVQLEASPAFLAVPVEK" was reported to be over-expressed in HCC sample [22]. This peptide was found to be over-expressed by Q-FISH, but under-expressed by spectral counting analysis by SEQUEST. The full list of peptides is given in Additional file 1. Based on this report, the 2 × 2 confusion tables can be constructed as shown in Table 3.

**Table 3:** 2 × 2 tables for literature search results of Q-FISH and SEQUEST

		Q-FISH			SEQUEST		
		
		HCC	Normal	Total	HCC	Normal	Total
Literature	Over-Expressed	**25**	17	42	**34**	26	60
	Under-Expressed	6	**17**	23	9	**24**	33

	Total	31	34	65	43	50	93

	Accuracy	64.62%			62.37%		

We assume that if a peptide is reported in a previous literature, it is assumed to be correctly identified. We compared two results (Q-FISH and SEQUEST) through literature search. Based on this report, the following 2 × 2 tables can be constructed

For Q-FISH result, 65 peptides were found in the literature: 31 for HCC sample and 34 for normal sample. Among 31 peptides for HCC sample, 25 are reported as over-expressed in the literature, and are assumed to be correctly identified. Among 17 peptides for normal sample, 17 are reported as under-expressed in the literature, and thus are assumed to be correctly identified. The remaining 17 and 6 peptides are assumed incorrectly identified.

For SEQUEST result, 93 peptides were reported in the literature: 43 for HCC sample and 50 for normal sample. Among them, 34 and 24 peptides were correctly identified, while 26 and 9 peptides were incorrectly identified. Based on these numbers, accuracy measure was computed showing that Q-FISH (accuracy = 64.62%) has slightly higher accuracy than SEQUEST (accuracy = 62.37%). This comparison showed that Q-FISH performed as reliably as SEQUEST, despite the comparison giving SEQUEST a natural advantage.

Table 4 provides a list of potential protein biomarkers. Q scores were calculated by averaging the correlation coefficient between moving averages over the reference spectrum and experimental spectra of the clustered spectral set. If it has a relatively high value, then the reference spectrum is well represented in the clustered spectral set.

**Table 4:** Lists of differentially expressed peptides in HCC and normal sample.

HCC sample							
**Related** **Cancer**	**Gene Name**	**Shogun Sequence**	**#(HCC)^a^**	**XCorr**	**Q Score**	**Protein Name**	**PMID^c^**

HCC	AKR1B10	IVENIQVFDFK	2	2.04	0.95	Aldo-keto reductase family 1 member B10	20388846
	ALB	DVFLGMFLYEYAR	2	2.04	0.96	Putative uncharacterized protein ALB	20658536
	ECHDC3	VIIISAEGPVFSSGHDLK	2	2.14	0.95	Isoform 1 of Enoyl-CoA hydratase domain-containing protein 3, mitochondrial	21495032
	EEF1A2	THINIVVIGHVDSGK	3	2.29	0.83	Elongation factor 1-alpha 2	18161050
	EEF2	AYLPVNESFGFTADLR	3	3.30	0.96	Elongation factor 2	18161940
	ENO1	FTASAGIQVVGDDLTVTNPK	33	2.51	0.61	Isoform alpha-enolase of Alpha-enolase	18813785
	FGG	EGFGHLSPTGTTEFWLGNEK	2	3.16	0,95	Isoform Gamma-B of Fibrinogen gamma chain	19596924
	FN1	SSPVVIDASTAIDAPSNLR	2	2.45	0.96	Isoform 1 of Fibronectin	16820872
	FTCD	EAQELSLPVVGSQLVGLVPLK	2	2.98	0.99	Isoform A of Formimidoyltransferase-cyclodeaminase	18571811
	GAPDH	WGDAGAEYVVESTGVFTTMEK	5	3.57	0.96	Glyceraldehyde-3-phosphate dehydrogenase	20714864
	HBD	FFESFGDLSSPDAVMGNPK	2	2.37	0.96	Hemoglobin subunit delta	9214599
	HMOX1	ALDLPSSGEGLAFFTFPNIASATK	2	2.82	0.90	Heme oxygenase 1	20664735
	HRSP12	IEIEAVAIQGPLTTASL	2	2.31	0.98	Ribonuclease UK114	18349270
	HSPA5	NQLTSNPENTVFDAK	4	2.51	0.97	HSPA5 protein	19445531
	HSPA9	VINEPTAAALAYGLDK	2	2.04	0.93	Stress-70 protein, mitochondrial	18334731
		DIVMTQSPDSLAVSLGER	2	2.52	0.99		
	HSPD1	ALMLQGVDLLADAVAVTMGPK	3	2.66	0.95	60 kDa heat shock protein, mitochondrial	21533669
	NME1	VMLGETNPADSKPGTIR	2	2.57	0.97	Isoform 1 of Nucleoside diphosphate kinase A	17594820
		EISLWFKPEELVDYK	2	2.27	0.95		
	P4HB	VDATEESDLAQQYGVR	2	2.38	0.81	Protein disulfide-isomerase	21207424
	PRDX6	LIALSIDSVEDHLAWSK	3	3.48	0.93	Peroxiredoxin-6	19893992
	TKT	ILATPPQEDAPSVDIANIR	3	2.16	0.98	cDNA FLJ54957, highly similar to Transketolase	17321041
	VCP	LIVDEAINEDNSVVSLSQPK	2	2.49	0.98	Transitional endoplasmic reticulum ATPase	12560433
	VIM	EMEENFAVEAANYQDTIGR	3	3.28	0.99	Vimentin	19843643

breast cancer	EEF1D	SLAGSSGPGASSGTSGDHGELVVR	2	3.17	0.93	Elongation factor 1-delta	17997862
	HBB	GTFATLSELHCDK	2	2.09	0.97	Hemoglobin subunit beta	20097481

colon cancer	ACTN1	GYEEWLLNEIR	3	2.03	0.99	Alpha-actinin-1	17898132
		ACLISLGYDVENDR	2	2.09	0.94		
	ATP5B	DQEGQDVLLFIDNIFR	2	2.58	0.98	ATP synthase subunit beta, mitochondrial	20080835
	HMGCS2	LMFNDFLSASSDTQTSLYK	3	2.87	0.93	Hydroxymethylglutaryl-CoA synthase, mitochondrial	16940161

colorectral cancer	ATP5A1	NVQAEEMVEFSSGLK	2	2.65	0.95	ATP synthase subunit alpha, mitochondrial	9261598
		EVAAFAQFGSDLDAATQQLLSR	3	2.88	0.87		

Leukemia	IDH1	SIEDFAHSSFQMALSK	2	2.53	0.97	Isocitrate dehydrogenase [NADP] cytoplasmic	21205756

pancreatic cancer	EPPK1	LLEAQIATGGVIDPVHSHR	2	2.64	0.97	epiplakin 1	18498355

lung cancer	FGB	DNENVVNEYSSELEK	3	2.57	0.97	Fibrinogen beta chain	20142248

cell migration.	FLNB	YAPTEVGLHEMHIK	2	2.02	0.97	Isoform 1 of Filamin-B	20110358

	XRCC5	YAPTEAQLNAVDALIDSMSLAK	5	3.60	0.94	ATP-dependent DNA helicase 2 subunit 2	
	AP1B1	LAPPLVTLLSAEPELQYVALR	2	2.81	0.99	Isoform A of AP-1 complex subunit beta-1	
	PLEC	AGTLSITEFADMLSGNAGGFR	2	2.16	0.89	Isoform 1 of Plectin-1	
	SDHAF2	PAPEIFENEVMALLR	3	2.41	0.93	Protein EMI5 homolog, mitochondrial	
	TUBA4A	AFVHWYVGEGMEEGEFSEAR	2	2.40	0.98	Tubulin alpha-4A chain	
		AVFVDLEPTVIDEVR	2	2.23	0.98		
	TYMP	DVTATVDSLPLITASILSK	3	2.84	0.93	Thymidine phosphorylase	
	UGP2	TLDGGLNVIQLETAVGAAIK	2	2.98	0.94	Isoform 1 of UTP--glucose-1-phosphate uridylyltransferase	
	TUBB	AILVDLEPGTMDSVR	2	1.97	0.95	Tubulin beta chain	
	TPI	VTNGAFTGEISPGMIK	2	2.52	0.95	Triosephosphate isomerase (Fragment)	
	Unknown	LFIGGLSFETTEESLR	2	2.64	0.97	Putative uncharacterized protein HNRNPA2B1	
		SVPTSTVFYPSDGVATEK	3	2.77	0.93	cDNA FLJ54957, highly similar to Transketolase	
		RHVFGESDELIGQK	2	2.09	0.96		
		VFSNGADLSGVTEEAPLK	2	2.24	0.90	PRO2275	

Normal sample							

Related Cancer	Gene Name	Shogun Sequence	#(normal)^b^	XCorr	Q Score	Protein Name	PMID^c^

HCC	A2M	VSVQLEASPAFLAVPVEK	2	2.36	0.93	Alpha-2-macroglobulin	18959789
		LLLQQVSLPELPGEYSMK	3	2.25	0.96		
	ACTA2	YPIEHGIITNWDDMEK	3	2.42	0.96	Actin, aortic smooth muscle	21214675
	ALB	RPCFSALEVDETYVPK	2	2.18	0.90	Putative uncharacterized protein ALB	20658536
	ALDH2	VAEQTPLTALYVANLIK	2	2.55	0.86	Aldehyde dehydrogenase, mitochondrial	20186752
	ALDH6A1	ENTLNQLVGAAFGAAGQR	2	2.46	0.89	Methylmalonate-semialdehyde dehydrogenase [acylating], mitochondrial	17786358
		LFIHESIHDEVVNR	2	2.61	0.96		
		VNAGDQPGADLGPLITPQAK	2	3.27	0.98		
	ALDOB	GILAADESVGTMGNR	3	2.40	0.85	Fructose-bisphosphate aldolase B	17786358
		ELSEIAQSIVANGK	2	2.32	0.96		
	ASL	INVLPLGSGAIAGNPLGVDR	3	3.18	0.76	Argininosuccinate lyase	19138817
	ASS1	NPWSMDENLMHISYEAGILENPK	2	2.74	0.96	Argininosuccinate synthase	20104527
	BHMT	ISGQEVNEAACDIAR	2	2.23	0.62	Betaine--homocysteine S-methyltransferase 1	19960509
		AGPWTPEAAVEHPEAVR	2	2.62	0.93		
	C5orf33	VATQAVEDVLNIAK	2	2.23	0.97	Isoform 2 of UPF0465 protein C5orf33	21495032
	CAT	GAGAFGYFEVTHDITK	2	2.17	0.78	Catalase	21324921
		FNTANDDNVTQVR	2	2.40	0.92		
	FGG	AIQLTYNPDESSKPNMIDAATLK	3	3.76	0.92	Fibrinogen gamma chain	17018627
	ETFA	LEVAPISDIIAIK	5	2.73	0.89	Electron transfer flavoprotein alpha-subunit	20515076
	CPS1	TVLMNPNIASVQTNEVGLK	3	2.42	0.99	Isoform 1 of Carbamoyl-phosphate synthase [ammonia], mitochondrial	12143053
		FLGVAEQLHNEGFK	3	2.67	0.97		
		AVNTLNEALEFAK	2	2.58	0.96		
		VLGTSVESIMATEDR	3	2.22	0.88		
		IEFEGQPVDFVDPNK	2	2.52	0.98		
		GLNSESMTEETLK	2	2.63	0.95		
	CYP3A7	EMVPIIAQYGDVLVR	2	2.37	0.80	Cytochrome P450 variant 3A7	17978482
	DCI	DADVQNFVSFISK	3	2.20	0.99	Isoform 1 of 3,2-trans-enoyl-CoA isomerase, mitochondrial	1903293
	ECHS1	ALNALCDGLIDELNQALK	2	3.33	0.98	Enoyl-CoA hydratase, mitochondrial	15492826
	EIF5	AMGPLVLTEVLFNEK	5	2.41	0.83	Eukaryotic translation initiation factor 5	19175833
	FBP1	LDVLSNDLVMNMLK	7	2.34	0.72	Fructose-1,6-bisphosphatase 1	19637194
	FH	SGLGELILPENEPGSSIMPGK	3	2.20	0.98	Isoform Mitochondrial of Fumarate hydratase, mitochondrial	1958270
		AAAEVNQDYGLDPK	3	2.23	0.97		
		IYELAAGGTAVGTGLNTR	2	2.24	0.97		
	FLNA	ASGPGLNTTGVPASLPVEFTIDAK	3	2.68	0.97	Isoform 2 of Filamin-A	21471709
	HPD	SQIQEYVDYNGGAGVQHIALK	2	2.99	0.98	4-hydroxyphenylpyruvate dioxygenase	8558370
	HSPA5	SQIFSTASDNQPTVTIK	2	2.16	0.97	HSPA5 protein	19445531
	KRT8	LKLEAELGNMQGLVEDFK	59	2.08	0.43	Keratin, type II cytoskeletal 8	18932288
	PBLD	VNTENLLQVENTGK	2	2.33	0.94	Phenazine biosynthesis-like domain-containing protein	20525558
	PDIA4	EVSQPDWTPPPEVTLVLTK	3	2.49	0.98	Protein disulfide-isomerase A4	19016532
	PEBP1	GNDISSGTVLSDYVGSGPPK	6	3.51	0.96	Phosphatidylethanolamine-binding protein 1	20739083
	PHB	NITYLPAGQSVLLQLPQ	3	2.56	0.86	Prohibitin	21318481
	PRDX6	ELAILLGMLDPAEK	4	2.00	0.94	Peroxiredoxin-6	19893992
	SELENBP1	NTGTEAPDYLATVDVDPK	2	2.06	0.96	cDNA FLJ55757, highly similar to Selenium-binding protein 1	21338716
	SORBS1	LTPVQVLEYGEAIAK	2	2.64	1.00	Isoform 9 of Sorbin and SH3 domain-containing protein 1	11374898
	SORD	LENYPIPEPGPNEVLLR	2	1.99	0.97	Sorbitol dehydrogenase	12848999
	STIP1	ALSVGNIDDALQCYSEAIK	2	2.54	0.97	Stress-induced-phosphoprotein 1	17627933
	TPI1	VAHALAEGLGVIACIGEK	2	3.35	0.99	Isoform 2 of Triosephosphate isomerase	18813785
	TXNDC5	ALAPTWEQLALGLEHSETVK	3	4.01	0.98	Thioredoxin domain-containing protein 5	16574106
	Vκ3	EIVLTQSPATLSLSPGER	2	2.97	0.97	Rheumatoid factor D5 light chain (Fragment)	15207089
	ADH1A	FSLDALITHVLPFEK	6	2.60	0.92	Alcohol dehydrogenase 1A	16054971
		ELGATECINPQDYK	2	2.15	0.94		
	ADH4	ISEAFDLMNQGK	4	2.95	0.94	Isoform 2 of Alcohol dehydrogenase 4	16054971
		GGVDFALDCAGGSETMK	3	3.25	0.96		
		FNLDALVTHTLPFDK	8	2.76	0.95		
		AAIAWEAGKPLCIEEVEVAPPK	3	2.71	0.99		
		DLHKPIQEVIIELTK	5	3.08	0.99		

prostate cancer	COL6A2	YGGLHFSDQVEVFSPPGSDR	2	2.33	0.86	Isoform 2C2A' of Collagen alpha-2(VI) chain	18353764
		LLTPITTLTSEQIQK	3	2.57	0.93		
		VAVVTYNNEVTTEIR	5	2.38	0.67		
		IEDGVPQHLVLVLGGK	2	2.01	0.86		
	RPS27A	TITLEVEPSDTIENVK	2	2.23	0.98	ubiquitin and ribosomal protein S27a precursor	15647830

breast cancer	EMILIN1	LVGSGLHTVEAAGEAR	2	2.47	0.96	EMILIN-1	16243817
	MYH9	NLPIYSEEIVEMYK	2	2.06	0.97	Isoform 1 of Myosin-9	18796164
		QLLQANPILEAFGNAK	3	2.80	0.86	Isoform 1 of Myosin-9	
		IAEFTTNLTEEEEK	13	2.29	0.65	Isoform 1 of Myosin-9	

colon cancer	ALDH1A1	GYFVQPTVFSNVTDEMR	3	3.18	0.97	Retinal dehydrogenase 1	21435460
	ATP5B	TVLIMELINNVAK	5	3.18	0.88	ATP synthase subunit beta, mitochondrial	20080835
	ETFA	AAVDAGFVPNDMQVGQTGK	2	2.13	0.98	Electron transfer flavoprotein subunit alpha, mitochondrial	16708797
		GTSFDAAATSGGSASSEK	6	2.53	0.86		
	ANXA6	GLGTDEDTIIDIITHR	2	2.48	0.98	annexin VI isoform 2	21137014

Leukemia	GLUD1	HGGTIPIVPTAEFQDR	2	2.48	0.98	Glutamate dehydrogenase 1, mitochondrial	19683518
	IDH2	LNEHFLNTTDFLDTIK	3	2.77	0.98	Isocitrate dehydrogenase [NADP], mitochondrial	21205756

gastic carcinoma	HIST4H4	TVTAMDVVYALK	2	2.03	0.96	Histone H4	19139817

colorectal cancer	RRBP1	TLQEQLENGPNTQLAR	2	2.74	0.88	Isoform 3 of Ribosome-binding protein 1	19425502

pancreatic cancer	ARG1	TGLLSGLDIMEVNPSLGK	4	2.71	0.91	Isoform 1 of Arginase-1	21347333
	CALM1	VFDKDGNGYISAAELR	3	2.50	0.93	Calmodulin	18852131
		EAFSLFDKDGDGTITTK	2	2.62	0.98		

ovarian cancer	HAAO	TQGSVALSVTQDPACK	2	2.56	0.91	Isoform 1 of 3-hydroxyanthranilate 3,4-dioxygenase	19724865

Lung cancer	ACY1	TVQPKPDYGAAVAFFEETAR	2	2.50	0.99	cDNA FLJ60317, highly similar to Aminoacylase-1	8394326

cell migration.	FLNB	LVSPGSANETSSILVESVTR	2	3.21	0.99	Isoform 1 of Filamin-B	19915675
	UGP2	ILTTASSHEFEHTK	2	3.30	0.93	Isoform 1 of UTP--glucose-1-phosphate uridylyltransferase	
		IQRPPEDSIQPYEK	4	2.38	0.95		
	ALDH4A1	EEIFGPVLSVYVYPDDKYK	3	3.34	0,95	Delta-1-pyrroline-5-carboxylate dehydrogenase, mitochondrial	
	COL14A1	HFLENLVTAFDVGSEK	3	2.39	0.77	Isoform 1 of Collagen alpha-1(XIV) chain	
	DCTN2	LLGPDAAINLTDPDGALAK	2	2.24	0.94	dynactin 2	
	EEF1B2	SPAGLQVLNDYLADK	3	2.86	0.84	Elongation factor 1-beta	
	GRHPR	IAAAGLDVTSPEPLPTNHPLLTLK	3	3.11	0.99	Glyoxylate reductase/hydroxypyruvate reductase	
	HSD17B10	VMTIAPGLFGTPLLTSLPEK	3	2.80	0.91	Isoform 1 of 3-hydroxyacyl-CoA dehydrogenase type-2	
	PCBD1	VHITLSTHECAGLSER	2	2.54	0.96	Pterin-4-alpha-carbinolamine dehydratase	
	PDIA6	GSTAPVGGGAFPTIVER	3	2.05	0.87	Isoform 2 of Protein disulfide-isomerase A6	
	PTGR1	HFVGYPTNSDFELK	2	2.24	0.93	Prostaglandin reductase 1	
		TGPLPPGPPPEIVIYQELR	7	2.56	0.96		
	TF	SAGWNIPIGLLYCDLPEPR	3	2.65	0.97	Serotransferrin	
		EDPQTFYYAVAVVK	4	2.49	0.92		
	unknown	PAHVVVGDVLQAADVDK	2	2.88	0.96	22 kDa protein	

HCC and normal sample							

Related Cancer	Gene Name	Shogun Sequence	#(HCC)^a ^/#(normal)^b^	XCorr	Q Score	Protein Name	PMID^c^

HCC	CPS1	MEYDGILIAGGPGNPALAEPLIQNVR	2/11	3.92	0.91	carbamoyl-phosphate synthetase 1	12143053
		SIFSAVLDELK	1/8	3.87	0.92		
		IAPSFAVESIEDALK	3/13	2.96	0.85		
		TAVDSGIPLLTNFQVTK	1/10	2.50	0.45		
	HBA1	VADALTNAVAHVDDMPNALSALSDLHAHK	1/8	3.67	0.93	Hemoglobin subunit alpha 1	20572306
		VGAHAGEYGAEALER	4/13	2.05	0.94		
	P4HB	ILFIFIDSDHTDNQR	10/15	2.88	0.49	Protein disulfide-isomerase	21207424
	HNRNPC	MIAGQVLDINLAAEPK	21/9	2.31	0.46	Heterogeneous nuclear ribonucleoprotein C (C1/C2), isoform CRA_b	20572306
	PGK1	VSHVSTGGGASLELLEGK	16/8	3.36	0.46	Phosphoglycerate kinase 1	19200351
	ACTB	DLYANTVLSGGTTMYPGIADR	10/3	3.25	0.96	Actin, cytoplasmic 1	16493704
	GSTA1	NDGYLMFQQVPMVEIDGMK	2/6	2.24	0.83	Glutathione S-transferase	20604928
	FABP1	SVTELNGDIITNTMTLGDIVFK	17/6	3.43	0.78	Fatty acid-binding protein	12245374
	CES1	EGYLQIGANTQAAQK	13/1	2.21	0.76	Isoform 1 of Liver carboxylesterase 1	19658107

Breast Cancer	LGALS7/LGALS7B	LVEVGGDVQLDSVR	19/1	2.35	0.65	Galectin-7/p53-induced gene 1 protein	20382700
	HBB	FFESFGDLSTPDAVMGNPK	39/67	2.72	0.74	Hemoglobin subunit beta	20097481
	MDH2	VDFPQDQLTALTGR	4/7	2.49	0.93	Malate dehydrogenase 2	19485423
	MYH9	LQQELDDLLVDLDHQR	9/15	2.54	0.56	Myosin, heavy polypeptide 9, non-muscle, isoform CRA_a	18796164

Ovarian cancer	PSMA2	YNEDLELEDAIHTAILTLK	3/5	4.48	0.84	Proteasome subunit alpha type-2	14960231

Lung cancer	AKR1A1	DPDEPVLLEEPVVLALAEK	3/5	3.16	0.63	Aldo-keto reductase family 1	17114299

Chromophobe renal cell carcinomas	ATP5H	NLIPFDQMTIEDLNEAFPETK	3/5	2.48	0.95	ATP synthase subunit d, mitochondrial	20440404

Leukemia	IGKC	VDNALQSGNSQESVTEQDSK	3/6	3.95	0.92	Ig kappa chain C region	12357370

	RPS7	TLTAVHDAILEDLVFPSEIVGK	5/3	3.92	0.92	40S ribosomal protein S7	

^a ^the number of spectral sets in HCC samples

^b ^the number of spectral set in normal samples.

^c ^the PubMed index for MEDLINE

Table 4 shows lists of DEPs in HCC sample, normal sample, and both samples. In HCC sample and normal sample, 57 and 115 reference spectra were identified by SEQUEST. Among these spectra, 29 and 59 peptides were known biomarkers for the human liver cancer. In both sample, we performed a beta-binomial test for finding out DEPs. The result shows that only 84 out of 1,571 reference spectra indicate heterogeneity of spectral counts between HCC and normal tissue samples. Among these 84 reference spectra, only 22 were identified by SEQUEST.

To find the potential biomarkers in each sample, we searched the reference spectra of clusters using SEQUEST. Consequently, we could find 50 and 95 peptides as the candidate biomarkers from HCC sample and normal sample, respectively, as shown Table 4. Among them, 24 peptides in HCC sample and 56 peptides in normal samples are known biomarkers for the human liver cancer. Also, 22 reference spectra among 84 DEPs were identified by SEQUEST. Among them, 13 peptides are known markers for the human liver cancer, too.

As shown in Table 4, carbamoyl-phosphate synthetase 1 (CPS1) are annotated by various sequences such as "MEYDGILIAGGPGNPALAEPLIQNVR" "SIFSAVLDELK", "TAVDSGIPLLTNFQVTK" and "GLNSESMTEETLK". These sequences are underexpressed in the HCC sample. Kinoshita et al. [23] performed differential gene display analysis (DGDA) to compare the intensities of polymerase chain reaction (PCR) products and evaluated the degrees of mRNA expression in HCC tissue samples and noncancerous hepatitis tissues. Subsequently, they confirmed that CPS1 is underexpressed. Specifically, CPS1 synthesizes carbamyl phosphate from bicarbonate, adenosine triphosphate (ATP) and ammmonia. A genetic mutation of CPS1 was identified as the source of hyperammonemia. In HCC tissue samples, underexpression of the CPS1 gene had been reported in rats, but the scientists' study was the first to result in such a finding for humans [23]. Heterogeneous nuclear ribonucleoprotein C (HNRNPC) annotated as "MIAGQVLDINLAAEPK" and actin, cytoplasmic 1 (ACTB) annotated as "DLYANTVLSGGTTMYPGIADR" were found to be over-expressed in the HCC sample [24,25]. On the contrary, glutathione S-transferase (GSTA1) annotated as "NDGYLMFQQVPMVEIDGMK" has been down-regulated in the human HCC sample [26]. Moreover, fatty acid-binding protein (FABP1) annotated as "SVTELNGDIITNTMTLGDIVFK", and Isoform 1 of Liver carboxylesterase 1 (CES1) annotated as "EGYLQIGANTQAAQK" are all characteristic of the HCC sample [27,28].

**Figure 4:**
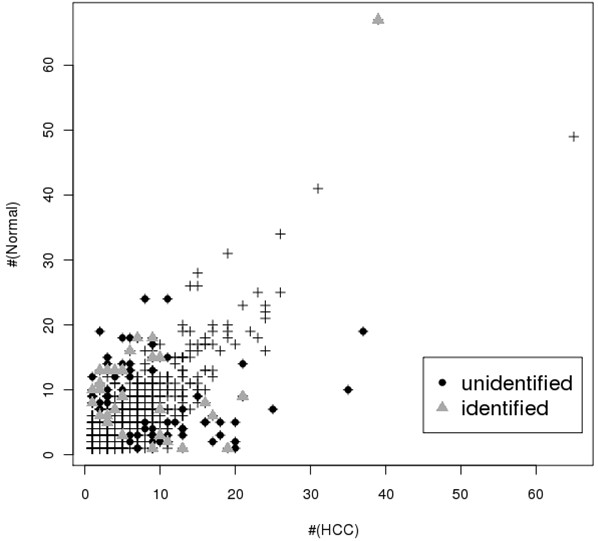
**Scatter plot of spectral counts between normal and HCC samples**. This figure plots the number of spectra in clustered sets in HCC and normal sample, respectively. The × axis and y axis represent the number of expressed spectra in each HCC and normal sample. Specifically, the grey triangle indicates DEPs identified with the use of SEQUEST, whereas the black circle indicates unidentified DEPs.

As shown in Table 4 many peptides are also known to be associated with cancer. Specifically, EMILIN-1 (EMILIN1), elongation factor 1-delta (EEF1D), galectin-7/p53-induced gene 1 protein (LGALS7), hemoglobin subunit beta (HBB) and malate dehydrogenase 2 (MDH 2) are differentially expressed in breast cancer cells [29-31]. Consequently, the LGALS7 gene is known to be related to over-expression when compared with control cells. Likewise, our result was also over-expressed. Table 4 provides a list of different types cancers associated with specific genes [28-34]. Figure 4 shows a scatter plot of the spectral counts of normal and HCC samples. The × axis and y axis represent the number of expressed spectra in each HCC and normal sample. Specifically, the symbol "▲" indicates DEPs identified with the use of SEQUEST, whereas the symbol "●" indicates unidentified DEPs. However, 62 DEPs were not identified by SEQUEST despite their significant differences by the beta-binomial test.

We believe there were several reasons why 62 DEPs were not identified by SEQUEST. First, "one-size-fits-all" search parameter values of SEQUEST would not have been chosen appropriately for this protein target. Second, these unidentified DEPs may have other post-translational modification, sequence variation (e.g., alternative splicing) or insufficient peptide ions information.

We re-run SEQUEST with many different parameter options for allowing phosphorylation modification and two missed cleavages, and for using other sequence databases (NCBI nr and EST human). However, even with these parameter options, SEQUEST did not identify the remaining 62 DEPs. Next, we tried to identify 62 reference spectra using other searching engines such as MASCOT and SpectraST. MASCOT identified 2 DEPs, Alcohol dehydrogenase 1A (ADH1A) and Isoform 2 of Myosin-9(MYH9) but SpectraST did not identify any DEPs. The remaining 60 DEPs could not be identified by these search engines. In order to identify these DEPs, further experiments may be needed. For example, additional MS/MS experiments such as MRM (Multiple Reaction Monitoring) or SRM (Selective Reaction Monitoring) can be carried out within the range of the corresponding retention times for all the unidentified spectra in order to collect more detailed peptide information.

## Conclusions

In this paper, we proposed a novel method to estimate peptide's abundance by counting MS/MS spectra clustered through the direct comparison of all experimentally observed spectra. For a given pair of spectra, our method can be used to answer the question of whether they are from the same peptide without computationally searching them from a theoretical library of protein spectra. Examining all possible pair-wise comparisons, our method results into a set of spectra for the same peptide and enables us to estimate the amount of peptides found in biological samples of interest by counting the spectra clusters. Since our proposed method compares all possible pairs of experimental spectra, it can discover even modified and unknown peptides, which may not be searchable from a theoretical spectral library. For practical MS/MS experimental data, a large proportion of spectra are often misidentified or completely lost during a computational database search. On the other hand, Q-FISH can identify these spectra without any loss of information. As demonstrated in our practical examples, the majority of DEPs derived by Q-FISH were found to be highly related with various cancers, which were not discovered by other methods.

We thus believe our Q-FISH algorithm will be highly useful in the identification of novel peptides [19]. Also, Q-FISH has the potential to find applications in many other practical proteomic studies. For example, it can be used to discover unknown biomarkers or drug targets through the comparison of proteins with statistically significant difference and by quantifying sets of identical peptides in multiple samples. Unknown spectral clusters can often come from non-peptide contaminants as revealed by a recent publication [35]. Q-FISH can evaluate the significance of such unknown clusters, some of which can be novel biomarkers, requiring further experimental confirmation by de novo sequencing, unrestricted sequence database search (using e.g. InsPect [36]) or spectral library search (using e.g. pMatch [37]).

## Methods

### Sample Preparation, Nano-LC-ESI-MS/MS

Tissue samples such as hepatocellular carcinoma (HCC) tumour tissue and adjacent healthy liver tissue were collected under the guidelines of the Institutional Review Board (IRB) established at Yonsei Medical Center (Seoul, Korea). All tissues were prepared and subsequently, in-solution tryptic digestion was performed as previously described [20]. Nano-LC-MS/MS analysis was performed on an Agilent Nano HPLC 1100 system using an linear trap quadruple (LTQ) mass spectrometer (Thermo Electron, San Jose, US). LC-MS/MS was performed as previously described [38]. The peptide fractionation was performed by means of cationic exchange chromatography (SCX) at a flow rate of 0.5 mL/min where absorbance of the column effluent was maintained stable at 280 nm for 40 min. Fractions were automatically transferred every 0.5 min into a 96-microplate.

Nano-LC MS/MS experiments were carried out three times on two different samples (human liver cancer and normal tissues) and 44,318 MS/MS spectra were generated. These tandem mass spectrometry data were first analyzed by means of the database search software SEQUEST (Bioworks 3.2, ThermoFinnigan, San Jose, US). The sequence database downloaded from European Bioinformatics Institute (EBI) was the International Protein Index (IPI) human version 3.61. The next step was to combine the protein sequence database with its reverse sequences. The maximum number of missed cleavage sites was set to 1, and only tryptic cleavage after arginine and lysine was allowed. The mass tolerance of the precursor peptide ion was set to 3.0 Da, while the fragment ion tolerance was set to 0.5 Da. These tolerance values were chosen to minimize FDR when XCorr > 1.5 [39]. Modification at cysteine with carboxyamidomethylation and methionine with oxidation were allowed [40]. All peptides assigned to reverse sequence were removed before proceeding to peptide identification to inhibit false-positive identifications. We chose XCorr as 1.44(+1), 1.97(+2) and 3.13(+3) which yielded FDR close to 0.05, respectively, and the value of DeltaCn is equal to a great than 0.1. These score criteria were considered to ensure high confidence in the results of protein identification [41]. The spectra derived by mass spectrometry were also analyzed by means of the spectral library search software SpectraST, which was initially developed by the Institute for Systems Biology (ISB) and National Institute of Standards and Technology (NIST). SpectraST is integrated with the Trans-Proteomic Pipeline (TPP) software suite, which provides the supporting functionalities necessary in a full proteomics data analysis pipeline. Then, the SpectraST program was validated in the NIST Human IT Library with the SpectraST's scores > 0.9 [18,38,42]. The precursor tolerance was set to 1.5 Da/z (Thomson).

### Q-FISH algorithm for direct comparison of experimental spectra

We assumed that MS/MS spectra from the same peptide would present similar patterns. Under this assumption, the proposed Q-FISH algorithm can be applied to find DEPs both in normal and disease samples. As shown in Figure 1, to evaluate the similarities between two spectra, we use a correlation coefficient of the moving window averages. The analytical process is summarized as follows:

#### 1. Scale Standardization

Perform scale standardization by dividing the intensity values by its maximum value.

#### 2. Moving average

Compute the moving window average over the spectra using a window of fixed size.

#### 3. Correlation index for moving average-based peak patterns

Calculate a summary statistic based on the correlation coefficient of the moving averages between two spectra.

#### 4. Spectral count-based quantification using two-stage clustering

Cluster duplicated peptides with similar peak patterns and retention time using a two-stage clustering method.

#### 5. Identification of differentially expressed peptides

Employ the beta-binomial test to identify DEPs among the experimental groups.

### Similarity measure between pairs of MS/MS spectra

#### Scale standardization

Because the intensities of the spectra obtained may be different for various physical and chemical reasons such as inconsistencies in the total ion currents, we cannot use the raw data for the intensity of m/z peaks. In light of this, we used a scale-standardization method, which involves the division of the m/z peak values for all ions by their maximum value. Let *x*[*i*] be the intensity of the *i^th ^*m/z peak. Then, the scale standardized intensity, *y*[*i*], is defined by

$$y\left[i\right]=\frac{x\left[i\right]}{\max\left(x\left[i\right]\right)}\;.$$

#### Moving window average

To reduce the background noise of the peak intensities, the moving window average (MWA) is used. The most simple moving average is the unweighted (or uniformly weighted) average of n data points within a given window, and the weighted moving average (WMWA) is the average calculated using multiplying weight factors to give different weight to each data point. Among the various options for the weights of WMWA, we selected the "Gaussian" kernel, which uses the probability density function (pdf) of the standard Gaussian distribution with mean 0 and variance 1 as a weight function.

For a given spectrum, the MWA is calculated by averaging the peak intensities within the sliding window sequentially for all m/z peaks. In other words, the MWA is not a single value, but a set of averages. The next step is to calculate correlation between the MWAs of two spectra and determine whether there are identical spectra from the same peptide.

We assume that there are *N *moving windows of fixed size *K *along the entire m/z range. Subsequently, the WMWA for the *i^th ^*moving window (*i *= 1, 2,..., *N*) is defined by

$$m\left[i\right]=\overset{K-1}{\underset{j=0}{\sum }}w_{j}y\left[i+j\right]\;,$$

where *y*[*i *+ *j*] is the *j^th ^*scale standardized intensity in the *i^th ^*moving window and *w_j _*are the weights. For a uniform kernel *w_j _= *1/*K *or the Gaussian kernel, *w_j _*= *Φ*(*z_j_*) represents the pdf of the standard Gaussian distribution, where *z_j _*represents the value of *y[i+j] *standardized by using mean and variance of m/z's in the *i^th ^*window. Total number of windows, *N *can be determined by the fixed window size *K *along with the entire m/z range (200-2000 Da). In order to determine the optimal window size, we randomly selected some pairs of spectra from the same and different peptides using target-decoy sequence database. We implemented receiver operating characteristic (ROC) analysis to determine the window size. Based on ROC analysis, we chose a window size, K = 30 (3.0Da) and accordingly N = 19,771 (20-2000 Da at interval of 0.1 Da). However, the areas under the curve (AUC) did not differ much and were less sensitive to the window size.

#### Correlation index for moving average-based peak patterns

For peptides *p *and *q*, the correlation coefficient is computed as follows:

$$r_{pq}=\frac{\sum_{i=1}^{N}\left(m_{p}\left[i\right]-{\bar{m}}_{p}\right)\left(m_{q}\left[i\right]-{\bar{m}}_{q}\right)}{\sqrt{\sum_{i=1}^{N}{\left(m_{p}\left[i\right]-{\bar{m}}_{p}\right)}^{2}}\sqrt{\sum_{i=1}^{N}{\left(m_{q}\left[i\right]-{\bar{m}}_{q}\right)}^{2}}}\;,$$

where ${\bar{m}}_{p}$and ${\bar{m}}_{q}$ are the means of moving window averages for peptide *p *and *q*. The closer the correlation coefficient is to 1, the stronger is the correlation between spectra from the same peptides.

### Quantification by counting spectra in clustered spectra set from a homogenous peptide

Two-stage cluster analysis is used to cluster peptide sets consisting of spectra with similar patterns. As previously assumed, if the spectra have approximately the same shape, then the spectra would have come from the same peptide. Namely, each cluster can be expected to be composed of the spectra obtained from a homogenous peptide. Two-stage clustering analysis employs two similarity measures to cluster peptides: the first is the difference between precursor ions and the second is the correlation coefficient between two MWAs. It is theoretically predicted that MS/MS spectra obtained from the same peptide have similar precursor ions. First, clusters can be defined in terms of pair-wise differences between the precursor ions. For any two pair of precursor ions in the same cluster, their difference is smaller than the threshold value. In our analysis, we set ± 1 Da as a threshold value. The next step is to perform a hierarchical clustering analysis for each of the clusters defined. Specifically, we employ "single linkage," also known as the nearest neighbour technique. Here, the correlation coefficient of MWAs is used as a similarity measure.

Because this two-stage clustering analysis yields clustered spectra sets consisting of MS/MS spectra from the same peptide, the amount of peptides can be quantified by counting the spectra included in each clustered set. Lastly, representative spectra called "reference spectra" can be defined based on the basic patterns of precursor ions as the average spectra for a given spectral set.

### Validation of the clustering results using retention times

It is well known that the same peptides tend to elute continuously within a limited liquid chromatography (LC) interval. Thus, the clustering results can be validated using the retention time (RT) information.

In order to validate the clustering results, we propose a new measure to estimate the clustering error rate using the spectral RT information. Note that the Q-FISH results provide the list of clusters. If a cluster contains only peptides from the same spectra, the RTs of peptides would have similar values. If a cluster contains peptides from the different spectra, the RTs would have different values. As a measure of similarity, we consider the measures representing the variability of RTs from the same cluster such as coefficient of variation (CV) and standard deviation (SD) of RTs. Since the RT varies much across of spectra, CV would be a better measure than SD. Using CV, we propose a new measure called the false clustering rate (FCR) which is similar in spirit to that of the false discovery rate (FDR). It measures the rate how often a cluster is composed of spectra from the different peptides. We provide a threshold value of CV, Δ, to determine whether a cluster is well clustered or not. That is, if the value of CV of a given cluster is smaller than Δ, then we call it is a good cluster. For the given value of Δ, FCR can be computed. The detailed procedure of computing FCR is given as follows:

1) Calculate the coefficient of variation (CV) of spectral RT in the same clusters from the Q-FISH results.

2) Permute the spectra while maintaining the number of spectra in each cluster fixed.

3) Calculate *CV_p _*for each permuted cluster for the *p*th permuted sample.

4) Compute FCR as follows:

$$FCR=\frac{\frac{1}{P}\sum_{p=1}^{P}\#\left\{i|CV_{p}\left(i\right)\leq \text{Δ}\right\}}{\#\left\{i|CV\left(i\right)\leq \text{Δ}\right\}},\;i=1,2,\cdots \;,C,$$

where *P *is the number of permutations, Δ the threshold value, and *C *the total number of clusters.

For our HCC data, we computed FCR for various values of Δ, as summarized in the Table 5. From our analysis, we chose the value of Δ as 4.4 which yielded FCR close to 0.05.

**Table 5:** Validation for clustering result using the false clustering rate (FCR)

FCR using RT information		FCR for the cut-off value	
**Δ**	**FCR**	**ρ**	***FCR***

1	0.0288	0.0	1.0000
2	0.0307	0.1	0.9486
3	0.0380	0.2	0.8060
4	0.0467	0.3	0.6525
**4.4**	**0.0500**	0.4	0.4515
5	0.0553	0.5	0.3178
6	0.0639	**0.6**	**0.0251**
7	0.0719	0.7	0.0034
8	0.0806	0.8	0.0008
9	0.0895	0.9	0.0003
10	0.0981	1.0	0.0000

In order to validate the clustering results, we propose a new measure to estimate the clustering error rate using the spectral retention time (RT) information. We computed the false clustering rate (FCR) for various values of threshold Δ, as summarized. We also calculated FCR to determine the cut-off value of correlation coefficient for spectral clustering. We computed *FCR *for the various values of the given ρ, as summarized. We chose ρ = 0.6 which yielded *FCR *close to 0.05.

We also calculated FCR to determine the cut-off value of correlation coefficient, ρ for spectral clustering. For the given threshold value of ρ, *FCR *can be computed in the similar manner as Δ. We computed *FCR *for the various values of the given ρ, as summarized in the Table 5. We chose ρ = 0.6 which yielded *FCR *close to 0.05.

### Differentially expressed peptides (DEPs)

To estimate the peptide's abundance found in different samples such as control and disease tissue samples, a spectral counting method like Q-FISH can be employed. Pham et al. [21] proposed the use of the beta-binomial distribution to test the significance of DEPs in spectral counts in label-free mass spectrometry-based proteomics. Their results revealed that the beta-binomial test can be applied to experiments with one or more replicates, as well as for the comparison of multiple conditions. We applied the beta-binomial model to test the abundance of DEPs in the clustered spectral set through three replicated MS/MS experiments.

Let *x *denote the number of spectral counts in the clustered spectral set and *n*, the total number of spectral counts of all spectral in each sample. Then, assume that *x *is distributed with the true proportion *π*, 0 ≤ *π *≤ 1,

x|π~Binomial(n,π)

Differently, *π *is approximated as a random variable based on the beta distribution with real parameters *α *> 0 and *β *> 0.

$$\pi \sim Beta\left(\alpha ,\beta \right),\;E\left(\pi \right)=\frac{\alpha }{\alpha +\beta }=\theta$$

$$Var\left(\pi \right)=\frac{\alpha \beta }{{\left(\alpha +\beta \right)}^{2}\left(\alpha +\beta +1\right)}$$

Subsequently, the marginal distribution of *x *is the beta-binomial distribution [21],

$$\begin{array}{ll} p\left(x|\alpha ,\beta ,n\right) & =\int_{0}^{1}p\left(x|\pi ,n\right)p\left(\pi |\alpha ,\beta \right)d\pi \;\\ & =\int_{0}^{1}\;\left(\begin{array}{c} n\\ x \end{array}\right)\frac{\pi^{x+\alpha -1}\left(1-\pi^{n-x+\beta -1}\right)}{B\left(\alpha ,\beta \right)}d\pi ,\;\\ & =\left(\begin{array}{c} n\\ x \end{array}\right)\frac{B\left(\alpha +x,n+\beta -x\right)}{B\left(\alpha ,\beta \right)}\;\\ & \; \end{array}$$

where *B*(·,·) is the beta function.

The following parameterization is used

$$\pi =\frac{\alpha }{\alpha +\beta }=h\left(Xb\right)=h\left(\eta \right)\;\text{and}\;\phi =\frac{1}{\alpha +\beta +1},$$

where *h *is the inverse of the link function (logit or complementary log-log), *X *a design matrix, *b *a vector of fixed effects, *η *= *Xb *the linear predictor, and *Φ *the overdispersion parameter. Based on this parameterization, the marginal mean and variance are:

$$E\left(x\right)=n\cdot \pi$$

$$Var\left(x\right)=n\cdot \pi \cdot \left(1-\pi \right)\cdot \left[1+\left(n-1\right)\cdot \phi \right].$$

It should be noted that parameters *b *and *ϕ *are estimated by maximizing the log-likelihood of the marginal model. Given the estimated coefficients, the testing hypothesis is rephrased as to whether the *b *coefficient is 0 [43]. We also used Benjamini and Hochberg's method to correct for multiple comparisons in multiple testing for DEPs [44].

## Competing interests

The authors declare that they have no competing interests.

## Authors' contributions

SML and MSK performed the statistical analysis and drafted the manuscript. HJL, YKP, and HT carried out mass spectrometry experiments. JKL and TP conceived of the study, and participated in coordination. All authors write, read and approved the final manuscript.

## Supplementary Material

Additional file 1 **Lists for identified peptides reported in the literature**. In order to compare the performance of Q-FISH with the spectral counting method by SEQUEST, we used the human liver data and validated the results through literature search. For the human liver data, Q-FISH provided 1571 differentially expressed clusters for HCC sample and 1556 for normal sample, among which 57 and 99 clusters were identified by SEQUEST in HCC and normal samples, respectively. On the other hand, SEQUEST provided 93 and 145 peptides for HCC and normal tissue samples, respectively. Click here for file
